# The ratio of serum neuron-specific enolase level to admission glasgow coma scale score is associated with diffuse axonal injury in patients with moderate to severe traumatic brain injury

**DOI:** 10.3389/fneur.2022.887818

**Published:** 2022-09-01

**Authors:** Weiliang Chen, Guanjun Wang, Chunyu Yao, Zujian Zhu, Rui Chen, Wen Su, Rongcai Jiang

**Affiliations:** ^1^Department of Neurosurgery, Tianjin Medical University General Hospital, Tianjin, China; ^2^Key Laboratory of Post-Neuroinjury Neuro-repair and Regeneration in the Central Nervous System, Tianjin Key Laboratory of Injury and Regenerative Medicine of Nervous System, Tianjin Neurological Institute, Ministry of Education, Tianjin Medical University, Tianjin, China; ^3^Department of Neurosurgery, Haining People's Hospital, Jiaxing, China

**Keywords:** traumatic brain injury, diffuse axonal injury, neuron specific enolase, early diagnosis, biomarker

## Abstract

**Background:**

Moderate to severe traumatic brain injury (TBI) is frequently accompanied by diffuse axonal injury (DAI). Considering the low sensitivity of computed tomography (CT) examination for microbleeds and axonal damage, identification of DAI is difficult using conventional diagnostic methods in the acute phase. Neuron-specific enolase (NSE) has been demonstrated to be increased in serum following various types of TBI and is already clinically/commercially available. We conjecture that serum NSE level to admission GCS score ratio (NGR) may be a useful indicator for the early diagnosis of DAI.

**Methods:**

This study included 115 patients with moderate-to-severe TBI who underwent NSE measurements within 6 h after injury and brain magnetic resonance imaging (MRI) within 30 days. The positive and negative DAI groups were divided according to MRI findings.

**Results:**

Among the 115 patients, 49 (42.6%) were classified into the DAI group and 66 (57.4%) patients into the non-DAI group by clinical MRI. The NGR of patients without DAI was found to be significantly lower than those of patients with DAI (*p* < 0.0001). NGR presented the largest Pearson *r* value (*r* = 0.755, 95% CI 0.664–0.824, *p* < 0.0001) and high diagnostic accuracy for DAI [area under the curve (AUC) = 0.9493; sensitivity, 90.91%; and specificity, 85.71%]. Patients with TBI presenting with higher NGR were more likely to suffer an unfavorable neurological outcome [6-month extended Glasgow Outcome Scale (GOSE) 1–4].

**Conclusions:**

The NGR on admission could serve as an independent predictor of DAI with moderate-to-severe TBI.

## Introduction

Moderate-to-severe traumatic brain injury (TBI) is one of the leading causes of death and neurological dysfunction (1, 2). About 62.9–72% of these patients were diagnosed with diffuse axonal injury (DAI) by magnetic resonance imaging (MRI) (3, 4). DAI is caused by acceleration-deceleration or rotational forces on brain tissues, resulting in axonal shear injuries and delayed axonal disconnection (5, 6). Patients suffering from DAI are often accompanied by a 6-h loss of consciousness, and the diagnosis and severity grading of DAI within the first 6 h may reduce the time to investigate the cause of the coma and provide opportunities for optimized treatment strategies (6, 7). The gold standard for diagnosing and grading DAI is histopathology, but for surviving patients, MRI is considered the primary basis for diagnosing DAI, because it shows subtle axonal damage and hemorrhages that cannot be detected by computed tomography (CT) (8). However, for patients with severe TBI who require multiple medical devices and drug treatments, lengthy queuing and examination times for MRI, coupled with the limitations of monitoring equipment due to high magnetic fields, hinder the application of MRI in the acute phase of injury (9). Therefore, the identification of DAI in the early stage of TBI is an urgent problem to be solved at present.

The role of biomarkers in the diagnosis and prognostic assessment of DAI has received increasing attention (9–11). Neuron-specific enolase (NSE) is an isoenzyme of the glycolytic enzyme enolase which exists almost in neuronal cells, with the highest concentrations in the central nervous system. The molecular weight of 78 KD facilitates the leakage of NSE into the extracellular compartment and blood after the structural damage of nerve cells (12). NSE has been demonstrated to be increased in serum following various types of TBI, making it potentially a sensitive and specific biomarker of neuronal cell damage (12, 13). However, small cell lung cancers, red blood cells, platelets, adipose tissue, and smooth muscle contain NSE as well (14).

The Glasgow Coma Scale (GCS) is recognized as one of the most important neurological tools for assessing neurological function in patients with TBI (15). The admission GCS score has been confirmed to be strongly associated with the severity and prognosis of patients with TBI, but it is susceptible to sedation or endotracheal intubation, and patients suffering from DAI may present with lower GCS scores in the acute phase, leading to an inability to accurately distinguish between the grade of DAI and long-term prognosis (15, 16).

In summary, the early diagnosis of DAI cannot be accomplished with a single index so far, although part of it can be reflected by serum NSE or admission GCS score. We conjecture that better evaluation performance may come from the combination of NSE and GCS. This study aimed to test the hypothesis that the serum NSE level to admission GCS score ratio (NGR) is an effective indicator for identifying DAI in the acute phase of injury.

## Materials and methods

### Study population

Between January 2019 and June 2021, patients with moderate-to-severe TBI were screened in this retrospective observational study at the emergency department (ED) of Haining People's Hospital of Zhejiang Province, which has 1,100 beds and provides medical services to the urban and surrounding areas and has more than 500,000 ED visits annually. This data collection site was approved by the local Institutional Review Board.

### Selection of participants

Patients admitted to the ED for moderate-to-severe blunt TBI within 6 h were enrolled in this study. Inclusion criteria were: patients of age of 18–80 years, having an admission GCS score ≤ 12, and serum NSE levels were measured together with other blood test samples in the ED. Reactions and quantification of NSE were performed with the Beckman DXI800 (Beckman Coulter, CA, USA), using a commercially available chemiluminescent immunoassay kit (Sichuan Orienter Biotechnology CO., Ltd. Chengdu, China). Exclusion criteria were: lack of MRI within 30 days of admission (21/51), pre-hospital sedation and intubation (17/51), progressive brain illness (Parkinson's disease, dementia, seizure disorder, multiple sclerosis, and brain tumor) (2/51), secondary brain injury (infarction, hemorrhage, and intracranial infection) (5/51), history of brain surgery or stroke without full recovery (1/51), severe peripheral organ complications have not been reversed (2/51), and TBI combined with severe circulatory failure (3/51).

### Parameters

Demographic and clinical characteristics were collected upon arrival to the ED, such as age, sex, causes of trauma (road traffic accident, fall, and others), admission GCS score, pupillary light reflex (none, unilateral, or bilateral), Marshall CT classification (evaluated on a scale from 1 to 6) from the first head CT scan, mean arterial blood pressure (MAP), hypotension, and hypoxia.

### Definitions

The clinical MRI was performed with a 1.5T scanner (Siemens Symphony, ATim). DAIs were defined as patients with moderate-to-severe TBI with lesions in the gray-white matter junction of the cerebrum, corpus callosum, or brain stem with T2-weighted imaging (T2WI), T2-weighted fluid attenuated inversion recovery (T2 FLAIR), and diffusion-weighted imaging (DWI) in magnetic resonance imaging (MRI) (9, 17). These lesions were defined as having hypointense focus presented on T2WI (hemorrhagic DAI), or hyperintense focus presented on DWI, T2 FLAIR, and T2WI (non-hemorrhagic DAI) (17, 18). The MRI and CT images were independently analyzed by two experienced neuroradiologists, who had access to the patient's clinical information but were blinded to the serum NSE levels.

The presence of DAI in the hemispheres or cerebellum was recorded as DAI grade 1, in the corpus callosum with or without lesions of grade 1 as DAI grade 2, and in the brainstem with or without lesions of grade 1 and/or 2 as DAI grade 3. Patients without DAI were assigned to grade 0 (4, 9).

### Outcome assessment

A functional neurological status assessment at 6 months post-injury was performed by a structured telephone survey of patients or caregivers. The extended Glasgow Outcome Scale (GOSE) was used to quantify 6-month outcomes as favorable outcomes (GOSE 5–8; no or moderate disability) or unfavorable outcomes (GOSE 1–4; severe disability or death). The GOSE was divided into 8 levels: 8: great recovery; 7: good recovery, and accompanied by minor physical and mental deficits; 6: moderately disabled, resuming the previous job but requiring some adjustments; 5: moderately disabled, qualified for low-level work; 4: severely disabled and can do some daily activities with the help of others; 3: severely disabled and totally dependent on others for daily activities; 2: plant state; and 1: death. The imputed 6-month GOSE average from the primary analysis in our study was assigned to those missing GOSE (DAI group 3/49 and non-DAI group 2/66) (19).

### Statistical analysis

All statistical analyses were performed using GraphPad Prism 8 (GraphPad Software, San Diego, CA, USA). A value of *p* < 0.05 with a two-tailed test was considered statistically significant. Categorical data are presented as frequency or percentage and compared by Fisher's exact test. Continuous data are presented as the median and interquartile range (IQR) and were compared by the Mann–Whitney *U*-test. The Pearson correlation analysis was employed to show the association of potential risk factors with DAI (the primary outcome variable), and presented in the form of a forest plot. The receiver operating characteristic (ROC) curve analysis was performed to assess the diagnostic accuracy of NGR on admission for identifying DAI after injury. The functional neurological outcome at 6 months post-injury for patients with moderate-to-severe TBI was compared as the secondary outcome variable.

## Results

### Enrollment and characteristics of the patients

**Figure 1:**
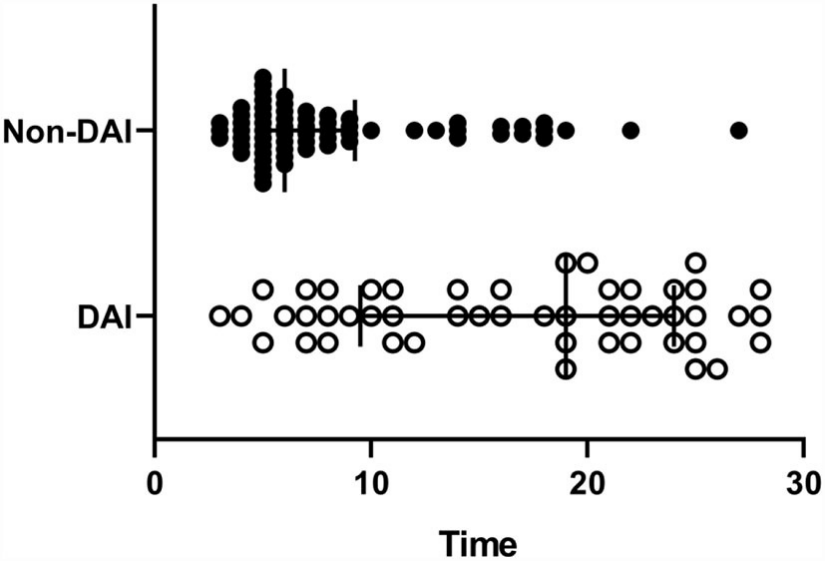
The timing of the MRI scans post-injury. The diffuse axonal injury (DAI) group was significantly longer than the non-DAI group (*p* < 0.0001), 19 (10–24) vs. 6 (5–9), and median [interquartile range (IQR)].

During the study period, 166 patients with TBI who met all the inclusion criteria were enrolled. Thus, 51 patients were excluded due to the exclusion criteria, and a total of 115 patients were analyzed. The clinical findings, injury-related variables of eligible patients are shown in Table 1. Of the 115 eligible patients, 81 patients (70.4%) were men and 49 patients (42.6%) were classified as the DAI group by MRI within 30 days of admission. The admission GCS scores were significantly lower in patients with DAI (*p* < 0.0001), as were the 6-month GOSE scores (*p* = 0.0056). Regarding sex, age, and cause of trauma, there were no significant differences between the DAI and non-DAI groups. The non-DAI group had significantly lower serum levels of NSE within 6 h after injury compared with the DAI group (*p* < 0.0001). The NGR of patients without DAI was found to be significantly lower than those of patients with DAI (*p* < 0.0001). The median time to MRI scans post-injury was 19 days in the DAI group, which was significantly longer than the 6 days in the non-DAI group (*p* < 0.0001) (Figure 1).

**Table 1:** Demographic and clinical characteristics of eligible patients with diffuse axonal injury (DAI) and non-DAI.

**Variables**	**DAI** **(*n* = 49)**	**Non-DAI (*n* = 66)**	***p*-value**
Male, *n* (%)	32 (65.3)	49 (74.2)	0.3105
Age (years), median (IQR)	64 (52–70)	56.5 (47–66)	0.0815
Cause of trauma, *n* (%)			
Road traffic accident	31 (63.3)	31 (47.0)	0.0922
Fall	17 (34.7)	30 (45.4)	0.2577
Others	1 (2.0)	5 (7.6)	0.2376
Pupillary light reflex, *n* (%)			
None pupillary light reflex	9 (18.4)	3 (4.6)	0.0278
Unilateral pupillary light reflex	18 (36.7)	11 (16.7)	0.0176
Bilateral pupillary light reflex	22 (44.9)	52 (78.8)	0.0003
GCS, median (IQR)	6 (4–7)	10 (9–12)	<0.0001
Marshall CT score, median (IQR)	5 (4–6)	4 (3–4.8)	<0.0001
NSE, median (IQR)	40.6 (34.1–47.6)	24.1 (18–31.4)	<0.0001
NGR, median (IQR)	7.4 (5.5–8.7)	2.6 (2–3.5)	<0.0001
6-month GOSE			
Favorable outcome, *n* (%)	17 (34.7)	58 (87.9)	<0.0001
Unfavorable outcome, *n* (%)	32 (65.3)	8 (12.1)	<0.0001

NSE, neuron-specific enolase; GCS, Glasgow Coma Scale; NGR, serum NSE level to admission GCS score ratio; GOSE, Extended Glasgow Outcome Scale; Favorable outcome, GOSE5–8; Unfavorable outcome, GOSE1–4; interquartile range (IQR) (25th−75th percentile).

### Relationship between risk factors and DAI

**Figure 2:**
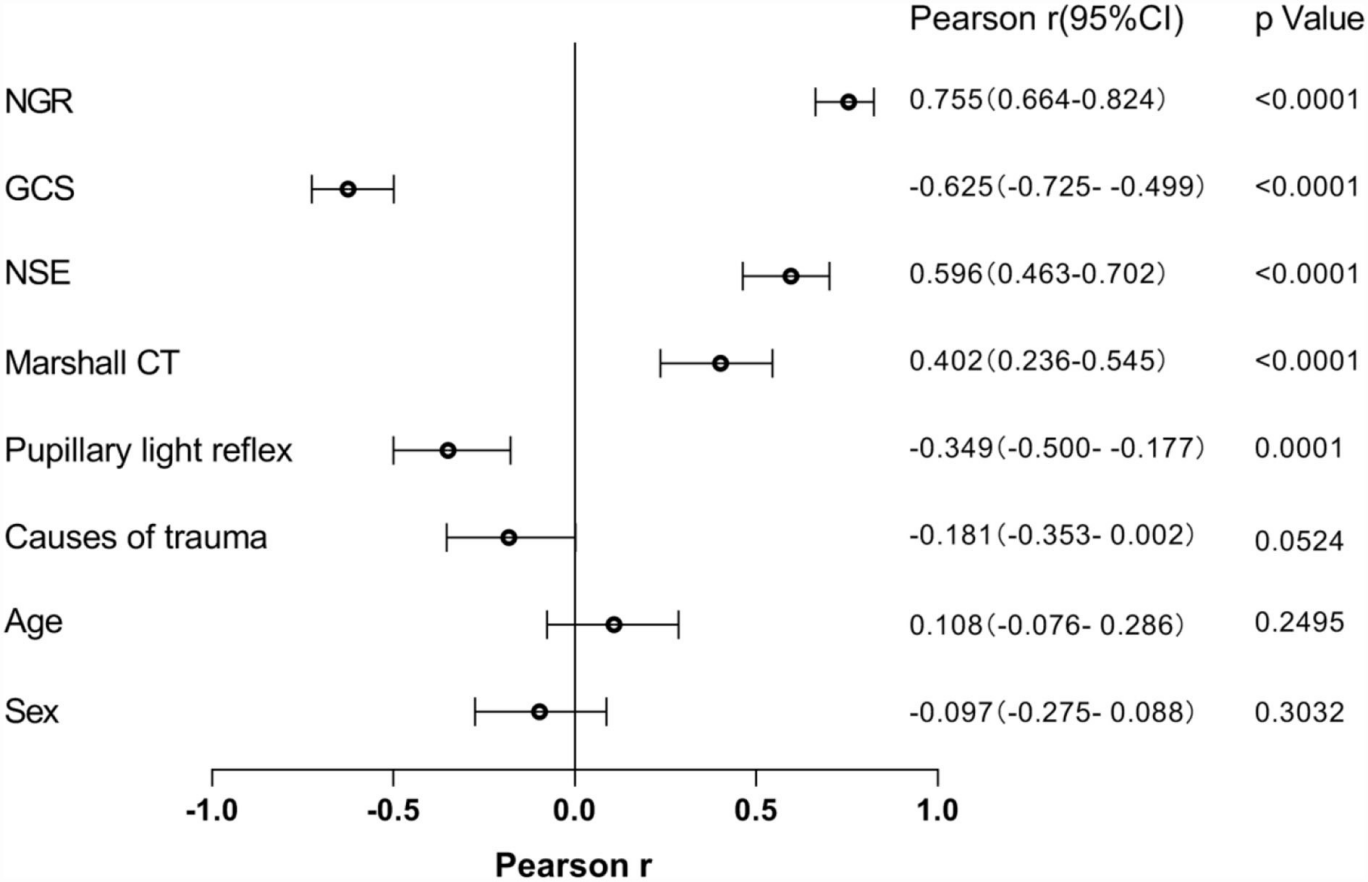
The relationship between potential risk factors with the presence of DAI on clinical MRI. NSE, neuron-specific enolase; GCS, Glasgow Coma Scale; NGR, serum NSE level to admission GCS score ratio.

The Pearson correlation analysis was employed to define the association of potential risk factors with the presence of DAI on clinical MRI (Figure 2). The following four factors were found to be negatively correlated with having DAI on MRI: GCS (*r* = −0.625), pupillary light reflex (*r* = −0.349), causes of trauma (*r* = −0.181), and sex (*r* = −0.097), of which GCS (*p* < 0.0001) and pupillary light reflex (*p* = 0.0001) were statistically significantly different. NGR (*r* = 0.755, *p* < 0.0001), NSE (*r* = 0.596, *p* < 0.0001), and Marshall CT (*r* = 0.402, *p* < 0.0001) were found to be significantly associated with an increased risk of the presentation of DAI after injury.

### Predictive power of significantly correlated factors for early diagnosis of DAI

**Figure 3:**
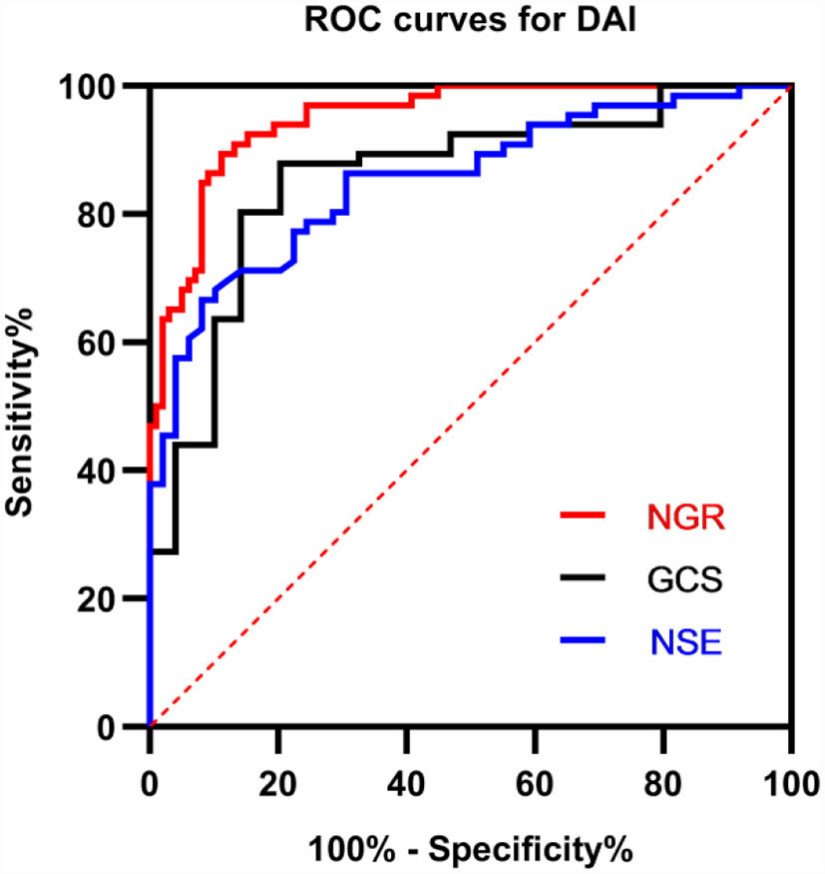
Receiver operator characteristic (ROC) curves for GCS, NSE, and NGR for early predicting DAI after injury. A larger probability result indicates better predictive power. The area under the curve (AUC) for NGR was 0.9493 (95% *CI* 0.9125–0.9861, *p* < 0.0001). NSE, neuron-specific enolase; GCS, Glasgow Coma Scale; NGR, serum NSE level to admission GCS score ratio.

The power of significantly correlated factors to predict DAI lesions on clinical MRI was further analyzed with the ROC curve, as shown in Figure 3. The area under the curves (AUCs) for NGR, NSE, and GCS were 0.9493 (95% CI 0.9125–0.9861, *p* < 0.0001), 0.8531 (95% CI 0.7853–0.9210, *p* < 0.0001), and 0.8575 (95% CI 0.7870–0.9279, *p* < 0.0001), respectively. With the best cut-off value of NGR (4.25), the sensitivity and specificity for early diagnosis of DAI were 90.91 and 85.71%, respectively.

**Figure 4:**
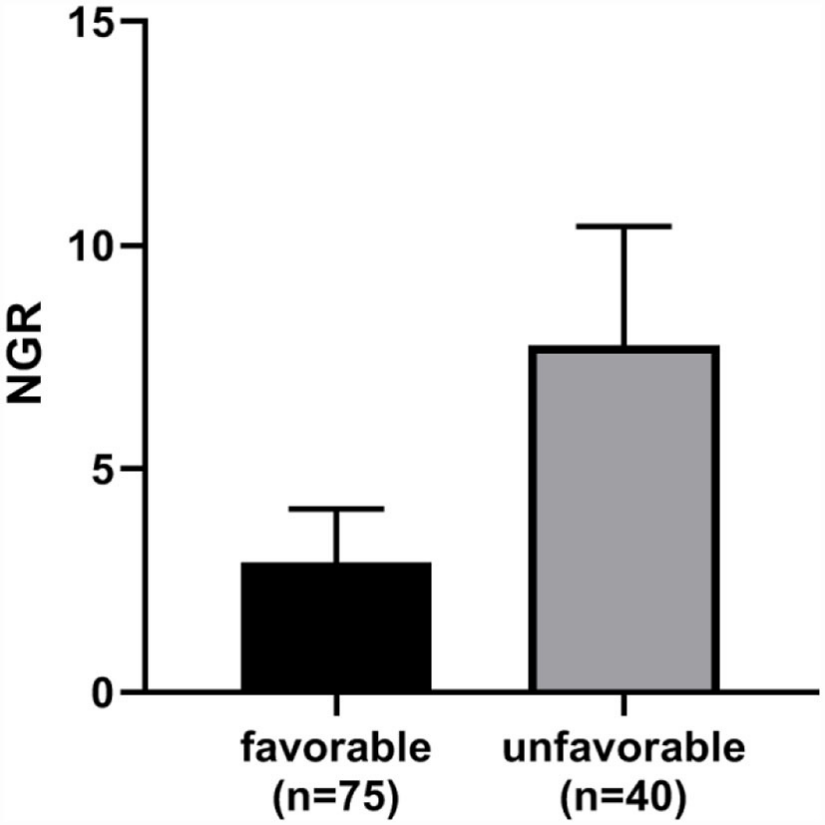
The NGR of the favorable outcome group was significantly lower than the unfavorable outcome group (*p* < 0.0001). Error bars indicate interquartile range. NGR, serum NSE level to admission GCS score ratio.

In addition, patients with TBI presenting with higher NGR were more likely to suffer an unfavorable neurological outcome (6-month GOSE 1–4) (Figure 4).

## Discussion

This retrospective study indicated that an increased value of NGR on admission could be employed as an independent predictor of DAI in patients with moderate-to-severe TBI. NGR demonstrated high diagnostic accuracy for DAI (AUC = 0.9493; sensitivity, 90.91%; and specificity, 85.71%). Patients with favorable neurological outcomes had significantly lower NGR as compared with those with unfavorable neurological outcomes.

Diffuse axonal injury is usually caused by external injury involving shearing force on brain tissues, and it manifests in the form of focal axonal shear injuries and axonal breakage (5, 6, 19). More than 50 million people suffer from TBI each year worldwide, among them, patients with moderate-to-severe TBI with DAI are more likely to have long-term sequelae or serious neurological deficits, even death (20–22). During initial resuscitation for TBI, effective and accurate prediction factors for DAI can contribute to support medical decisions for physicians and caregivers, from diagnosis to interventions (6, 9).

In recent years, many DAI biomarkers have been discovered by previous studies, such as tau protein, β-APP, NSE, S-100 calcium-binding protein B(S-100B), Ubiquitin C-terminal hydrolase-L1 (UCH-L1), spectrin, glial fibrillar acidic protein (GFAP), and neurofilament light (NfL) (5, 9, 18, 19, 23–26). GFAP and UCH-L1 have been shown to discriminate patients regarding the presence or absence of brain lesions on initial CT scan (26) and authorized for clinical use in the evaluation of mild TBI. Furthermore, Abbott has developed a rapid version on the i-STAT™ Alinity™ platform which received FDA approval in 2021 (27). We expect this device to be widely used in China and other countries to improve the diagnostic efficiency of DAI. Positive immunohistochemistry for β-APP is undetectable in normal brain tissue samples, whereas the immune response is enhanced starting 2 h after TBI. Following DAI, the disruption of axoplasmic transport leads to the accumulation of β-APP in axons, bringing its concentration to detectable levels (24). As the main cytoskeletal components of nerve cells, NfL and spectrin play important roles in maintaining axonal caliber and neuron morphology. Increased levels of these biomarkers result from axonal cell damage (28). Unfortunately, these biomarkers above are currently considered as the diagnostic indicators for DAI in laboratory settings and forensics and are not widely used in the management of patients with TBI (24). Tau protein, which accounts for a large proportion of microtubule-associated proteins, is released in great quantities into the cerebrospinal fluid after patients suffered DAI. Therefore, the content of tau protein in cerebrospinal fluid can be regarded as an indicator for quantitative evaluation of the severity of axonal injury. However, the tau protein detected in serum has not been found to be helpful for effectively evaluating prognosis. As a result, it is not enough to be used as an early diagnostic indicator of DAI in emergency treatment (24). S-100B and NSE have been investigated as potential biomarkers for predicting early neurological outcome or mortality. However, as independent predictors, the results are unsatisfactory. In fact, these molecules in tissues other than brain tissue severely limit their application, since serum levels of these biomarkers may also be increased in the serum of trauma patients without TBI (29, 30).

All hospitals above second-level in China have set up trauma treatment centers in EDs, all of which are able to treat patients with TBI. However, the detection capabilities of each hospital are inconsistent, and many new inspection and testing methods are only routinely implemented in third-level hospitals. Therefore, the diagnostic capability of DAI after injury is currently very limited. This study was dedicated in exploring the use of conventional testing methods combined with clinical characteristics to improve the diagnostic ability of DAI and avoiding the impact of missed diagnosis or misdiagnosis on the formulation of treatment strategies. By analyzing the demographic and clinical characteristics of eligible patients with TBI, no significant differences were found between patients with and without DAI with respect to sex, age, and cause of trauma. The non-DAI group had significantly lower serum NSE levels, Marshall CT score, and NGR compared with the DAI group (*p* < 0.0001). The admission GCS scores were significantly lower in patients with DAI (*p* < 0.0001). These conclusions are consistent with previous research findings (6, 19, 31). Considering the low sensitivity of CT examination for microbleeds and axonal damage caused by DAI in the acute phase (32, 33). Additionally, serum NSE is widely used by medical institutions at all trauma treatment centers in China because of its advantages of being easily accessible, low-cost (30–40 RMB), objective, and repeatable (12, 34, 35). We excluded Marshall CT score and combined serum NSE levels within 6 h of injury and admission GCS scores to form a simple, objective, and stable predictor.

In a study enrolling 176 patients, 106 patients were assigned to the non-DAI group and 70 were assigned to the DAI group. Logistic regression analysis identified that the GCS and Rotterdam CT scores were strongly related to the presence of DAI on subsequent MRI, with adjusted odd ratios (*OR*s) of 0.877 and 1.047, respectively (18). In our study, the correlation of admission GCS scores and DAI was also proved by the Pearson correlation analysis (*r* = −0.625, *p* < 0.0001), as did Marshall CT (*r* = 0.402, *p* < 0.0001). The strong correlation of the variable with DAI was shown by the large absolute value of Pearson *r*, with NSE (*r* = 0.596, *p* < 0.0001) being more significant. However, the largest Pearson *r* value was exhibited by NGR (*r* = 0.755, 95% CI 0.664–0.824, *p* < 0.0001), indicating that NGR was most correlated with the presence of DAI on clinical MRI.

Serum levels of NSE at admission were related to initial GCS scores and 6-month Glasgow Outcome Scale scores. Thus, serial measurements of serum NSE may help assess brain damage. Park et al. analyzed data concerning pediatric patients with TBI (36). According to Skandsen et al., DAI was found in nearly three-quarters in patients with moderate and severe TBI in the acute phase and GCS was correlated to the outcome only if patients with TBI had accompanying DAI (4). To further confirm the predictive power of significantly correlated factors for an early diagnosis of DAI, ROC curves were employed to assess the diagnostic accuracy of NGR, NSE, and GCS on admission for identifying DAI after injury. The final results showed that NGR had the highest diagnostic accuracy (AUC = 0.9493; sensitivity, 90.91%; and specificity, 85.71%) compared with GCS and NSE.

Based on a feature selection method using a tree-based ensemble algorithm, GCS, age, glucose, and fibrin/fibrinogen degradation products were identified as the effective prognostic factors for poor in-hospital outcome. Matsuo et al. analyzed data from 232 patients with TBI (37). Our study found that NGR was a more convenient and practical indicator for predicting 6-month GOSE because of its advantages of being easily accessible and objective.

## Limitations

There are several limitations to our present study. First, the sample size is not large enough to evaluate the predictive value of NGR for the occurrence of DAI after injury. Second, the pathological process after TBI is relatively complex, and there are many differential metabolites in the blood (38, 39), but only the serum NSE levels combined with GCS on admission were employed to predict the appearance of DAI and 6-month GOSE. Plasma neurofilament light (NfL), glial fibrillary acidic protein (GFAP), and ubiquitin C-terminal hydrolase-L1 **(**UCH-L1) will be considered for inclusion in predictive models in expanded studies after they are widely used in routine clinical practice in China. Third, NSE levels are time-dependent factor (12, 35), but in our study, the effect of dynamic changes of NSE levels on the 6-month prognosis of moderate to severe TBI was not further evaluated. Fourth, serum of trauma patients with TBI cannot fully reflect the pathological process of brain tissue injury in the early stage, which may bias the hyper-acute assessment.

## Conclusion

Our study demonstrated that NGR on admission could serve as an independent predictor of DAI with moderate-to-severe TBI and the best cut-off value of NGR on admission was 4.25 with a sensitivity of 90.91% and a specificity of 85.71%. This indicates that NGR is an easily accessible, objective, and reliable indicator to predict DAI in the acute phase of injury.

## Data availability statement

The original contributions presented in the study are included in the article/Supplementary material, further inquiries can be directed to the corresponding author.

## Ethics statement

The studies involving human participants were reviewed and approved by Ethics Review Committee of Haining People's Hospital. The patients/participants provided their written informed consent to participate in this study. Written informed consent was obtained from the individual(s) for the publication of any potentially identifiable images or data included in this article.

## Author contributions

RJ and WC contributed to the initial idea for this study. WC, RJ, and GW completed the study design. ZZ, RC, and CY developed and revised the search strategy. WS and GW contributed to consults about clinical issues. WS and RC extracted the data. WC analyzed the extraction data. RJ, CY, GW, and WC contributed to the revision of the draft. All authors approved the final manuscript prior to submission.

## Funding

This study was supported by the Tianjin Research Program of Application Foundation and Advanced Technology (grant 19YFZCSY00650).

## Conflict of interest

The authors declare that the research was conducted in the absence of any commercial or financial relationships that could be construed as a potential conflict of interest.

## Publisher's note

All claims expressed in this article are solely those of the authors and do not necessarily represent those of their affiliated organizations, or those of the publisher, the editors and the reviewers. Any product that may be evaluated in this article, or claim that may be made by its manufacturer, is not guaranteed or endorsed by the publisher.
